# A paradox of epidemics between the state and parameter spaces

**DOI:** 10.1038/s41598-018-25931-6

**Published:** 2018-05-14

**Authors:** Hengcong Liu, Muhua Zheng, Zonghua Liu

**Affiliations:** Department of Physics, East China Normal University, Shanghai, 200062 China

## Abstract

It is recently revealed from amounts of real data of recurrent epidemics that there is a phenomenon of hysteresis loop in the state space. To understand it, an indirect investigation from the parameter space has been given to qualitatively explain its mechanism but a more convincing study to quantitatively explain the phenomenon directly from the state space is still missing. We here study this phenomenon directly from the state space and find that there is a positive correlation between the size of outbreak and the size of hysteresis loop, implying that the hysteresis is a nature feature of epidemic outbreak in real case. Moreover, we surprisingly find a paradox on the dependence of the size of hysteresis loop on the two parameters of the infectious rate increment and the transient time, i.e. contradictory behaviors between the two spaces, when the evolutionary time of epidemics is long enough. That is, with the increase of the infectious rate increment, the size of hysteresis loop will decrease in the state space but increase in the parameter space. While with the increase of the transient time, the size of hysteresis loop will increase in the state space but decrease in the parameter space. Furthermore, we find that this paradox will disappear when the evolutionary time of epidemics is limited in a fixed period. Some theoretical analysis are presented to both the paradox and other numerical results.

## Introduction

Epidemic spreading has been well studied in the last two decades and its main attention has been focused on the influence of network topologies^1–3^. These studies involve almost all parts of epidemics such as the infinitesimal threshold^4–9^, reaction-diffusion model^10–13^, flow driven epidemic^14–18^, objective spreading^19,20^, temporal and/or multilayered networks^21–30^, and other aspects^31–39^ etc. Notice that an epidemic outbreak constitutes of both the growing and recovering processes. A common point of these studies is that their dynamics are only focused on the growing process, while little attention has been paid to the recovering process. Does it mean that the recovering process can be considered only as an inverse process of the growing process and thus there is no necessary to study it? To confirm this argument, amounts of real data of recurrent epidemics have been rechecked recently^40,41^. It is found that for all the outbreaks in real data, their growing and recovering processes are asymmetric and thus form a phenomenon of hysteresis loop^42^, indicating that the recovering process is not a simple inverse process of the growing process.

To understand the mechanism of hysteresis loop, we now make an analysis on these real data and find their two features. The first one is that the period of an epidemic outbreak generally takes a couple of months or even longer. During this relatively long period, the infectious rate *β* will gradually increase and then decrease due to whether, humidity and other factors, i.e. being seriously influenced by the seasonal variation^43–50^. The second one is that the infectious process will keep going when the value of *β* is updated to a new one, i.e. an adiabatical process where the final state of system with the last *β* is used as the initial state of system with the updated *β*. In this sense, a model based on adiabatical increase of infectious rate *β* has been recently proposed to reproduce the hysteresis loop^42^, where the initial conditions of infected seeds at each updated *β* are inherited from the final state of system with the last *β*. This way of adiabatically changing *β* is completely different from the previous studies where *β* is allowed to be updated only when system reaches its stationary state and the initial infected seeds for each updated *β* are always reset randomly^1–3^. The consequence is that the former results in a hysteresis loop, while the latter has no hysteresis loop. Then, an interesting question is what is the relationship between these two approaches, i.e. how can we make a transition between them. On the other hand, the hysteresis loop in ref.^42^ is observed in parameter space but not in state space or data space. Considering that the collected epidemic data are from the state space but not from the parameter space, it will be definitely more convincing if we can directly explain the data from the state space, in contrast to explain them indirectly from the parameter space in ref.^42^. Moreover, it would be necessary to understand why the sizes of epidemic outbreaks in real data are different from one to another.

In this work, we address these questions directly from the state space, in contrast to the hysteresis loop indirectly observed in parameter space in ref.^42^. We first study how the infectious rate increment Δ*β* and transient time *T* at each updated *β* influence both the size of hysteresis loop and the size of epidemic outbreak. We numerically find that both of these two parameters have significant influence to the transition between the status with hysteresis loop and that without hysteresis loop. For fixed Δ*β*, there is a critical *T*_*c*_ where the system will have a hysteresis loop when *T* < *T*_*c*_ but no hysteresis loop when *T* > *T*_*c*_. While for fixed *T*, there is also a critical Δ*β*_*c*_ where the system will have a hysteresis loop when Δ*β* Δ*β*_*c*_ but no hysteresis loop when Δ*β* < Δ*β*_*c*_. Thus, the size of hysteresis loop is mainly determined by the matching between Δ*β* and *T*. Then, we compare the results between the state and parameter spaces and interestingly find a contradictory dependence of the size of hysteresis loop on the parameters Δ*β* and *T* in the two spaces when the evolutionary time of epidemics is long enough, indicating that there is a paradox between the two spaces. To understand it, we provide a theoretical formula to unify this paradox. Further, we show that the paradox will disappear when the evolutionary time of epidemics is limited in a fixed period. Moreover, a theoretical analysis is presented to explain the numerical results.

## Results

### Hysteresis loop in state space

**Figure 1:**
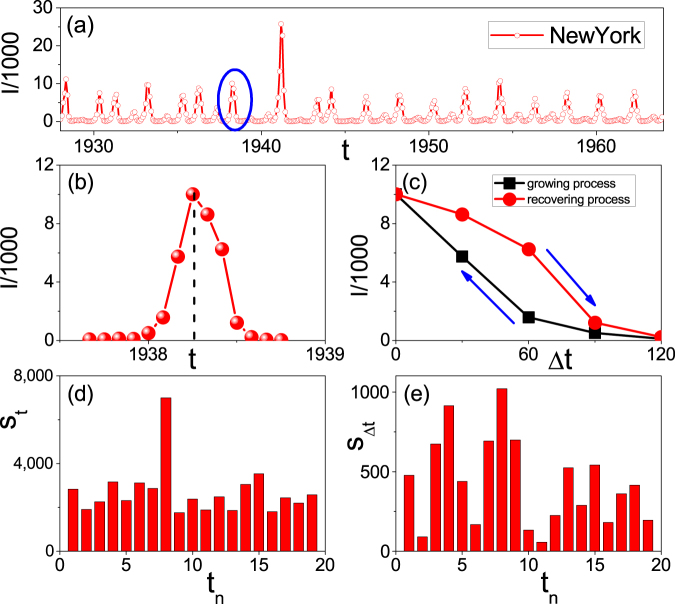
A typical real data of recurrent epidemics and its features. (**a**) Represents the time series of reported measles infective cases *I* in New York, where the variable *I* is from 0 to 3 × 10^4 ^^41^. (**b**) Amplification of the outbreak marked in the blue circle of (**a**). (**c**) The hysteresis loop of (**b**) in the rescaled framework where the original point is taken as the time pointed by the dashed line in (**b**) and the “squares” and “circles” denote the growing and recovering phases, respectively. (**d**) The area *S*_*t*_ of each outbreak in (**a**) where *t*_*n*_ is the number of outbreaks. (**e**) The area *S*_Δ*t*_ of hysteresis loops for the successive outbreaks in (**a**).

A characteristic feature of recurrent epidemic data is its multiple peaks or outbreaks surrounded by small amplitude backgrounds. Such examples can be found in many real data such as the data from Hong Kong, New York and Baltimore etc^40,41^. Figure 1(a) shows one of them from New York. Recently, ref.^42^ revealed that most of the outbreaks in Fig. 1(a) are asymmetric and can be illustrated by Fig. 1(b) for the typical one marked by the blue circle in Fig. 1(a). We see that the outbreak consists of both the growing and recovering processes, separated by the “dashed” line. It is easy to notice that the two processes are asymmetric. Letting the time *t*_0_ of the peak in Fig. 1(b) be the original point and Δ*t* = |*t* − *t*_0_| be the rescaled time, the asymmetry can be seen more clear by Fig. 1(c) in the rescaled time where the “squares” and “circles” denote the growing and recovering processes, respectively. We see that the two processes constitute a hysteresis loop, marking the asymmetry between the growing and recovering processes. Ref.^42^ pointed out that this kind of hysteresis loop exists in all the outbreaks of Fig. 1(a) and other recurrent epidemic data (not shown here), and can be understood in parameter space.

To go deep into the underlying mechanism, it is better to explain the phenomenon of hysteresis loop directly from state space, in contrast to that from parameter space in^42^. For this purpose, we introduce *S*_*t*_ and *S*_Δ*t*_ to represent the areas surrounded by the growing and recovering processes in Fig. 1(b) and (c), respectively. Figure 1(d) and (e) show the values of *S*_*t*_ and *S*_Δ*t*_ for the successive outbreaks in Fig. 1(a), respectively, where *t*_*n*_ represents the number of successive outbreaks. From Fig. 1(d) we see that *S*_*t*_ is different for different *t*_*n*_, indicating that the size of *S*_*t*_ is seriously influenced by some key factors such as the seasonal weather, humidity and sunlight etc. From Fig. 1(e) we see that all the *S*_Δ*t*_ are different and not zero, indicating that the existence of hysteresis loop is a general phenomenon in recurrent epidemics.

To figure out the key quantities influencing the sizes of *S*_*t*_ and *S*_Δ*t*_, we here adopt the model of reproducing the hysteresis loop in ref.^42^, which is in fact a susceptible-infected-susceptible (SIS) model with varying *β*. We notice from Fig. 1(a) that each epidemic outbreak lasts a relatively long time, i.e. a few months, marking the seasonal variation. On the other hand, the data in Fig. 1(a) is not from one or a few initial seeds in the same initial stage but a sum from different initial seeds at different initial stages. Thus, *β* reflects most probably the influence of environment, i.e. a match of whether, humidity and other factors^43–50^. In this sense, we may assume that the growing process corresponds to the gradual increase of *β* from 0 to *β*_*max*_, while the recovering process corresponds to the gradual decrease of *β* from *β*_*max*_ to 0. According to^42^, the increase or decrease of *β* is not continuous but discrete with an increment Δ*β*. For each updating of *β*, the initial conditions for the system with *β* + Δ*β* will be inherited from the last values of state variables at the previous *β*, called *adiabatically increase of β*. Based on the experience observation that we generally have a few continuously sunny days or raining days in a season, we let *T* be the time period for a *β* to remain unchanged. Therefore, *β* will be updated as follows

1 $$\begin{array}{rcl}\beta (t+\mathrm{1)} & = & \beta (t),\,if\,t\ne nT,\,n=\mathrm{1,}\,\mathrm{2,}\ldots ,\\ \beta (t+\mathrm{1)} & = & \beta (t)\pm {\rm{\Delta }}\beta ,\,if\,t=nT,\,n=\mathrm{1,}\,\mathrm{2,}\ldots \end{array}$$

where “+” and “−” correspond to the growing and recovering processes, respectively.

In numerical simulations, we initially choose a small value of *β* and a few infected seeds. Then, we let the system freely run a period of time *T* where a susceptible individual will become infected with probability *β* if he/she is connected to an infected neighbor and an infected one will recover to susceptible again with probability *μ*^1^. When there are more than one infected neighbors, a susceptible individual will become infected with probability $1-{\mathrm{(1}-\beta )}^{{k}_{inf}}$ where *k*_*inf*_ is the number of its infected neighbors. After a time period of *T*, we let *β* have an increase as in Eq. (1) but keep the individual states unchanged. We repeat this process until *β* reaches its maximum *β*_*max*_. After that, we simulate the recovering phase by letting *β*(*t* + 1) = *β*_*max*_ − Δ*β* but remain the individual states. Then, we let the system run as a traditional SIS model. Once *t* = *nT* for *n* = 1, 2, …, we let *β* have a decrease as in Eq. (1) and keep all the other aspects unchanged. We repeat this process until *β* reaches zero.

**Figure 2:**
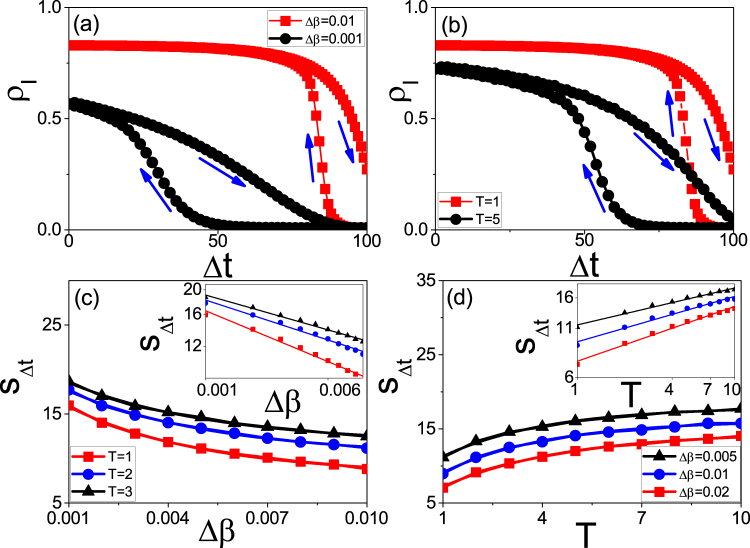
Hysteresis loops in state space where the arrows denote the evolutionary directions. (**a**) *ρ*_*I*_ versus Δ*t* for fixed *T* = 1 where the curves with “squares” and “circles” represent the cases of Δ*β* = 0.01 and 0.001, respectively. (**b**) *ρ*_*I*_ versus Δ*t* for fixed Δ*β* = 0.01 where the curves with “squares” and “circles” represent the cases of *T* = 1 and 5, respectively. (**c**) *S*_Δ*t*_ versus Δ*β* where the curves with “squares”, “circles” and “triangles” represent the cases of *T* = 1, 2 and 3, respectively. The inset shows the log-log plot. (**d**) *S*_Δ*t*_ versus *T* where the curves with “squares”, “circles” and “triangles” represent the cases of Δ*β* = 0.02, 0.01 and 0.005, respectively. The inset shows the log-log plot.

We take the random Erdős-Rényi (ER) network with size *N* = 10000 and average degree 〈*k*〉 = 6 as an example^51^. We fix *μ* = 0.2 in this paper and study how the parameters Δ*β* and *T* influence the size of hysteresis loop. Firstly, we consider the case of fixing *T* = 1. We let the growing process evolve to time *t* = 100 and then let the system turn to the recovering process until *t* = 200. Doing the same as in Fig. 1(c), we let the time *t* = 100 be the original point *t*_0_ and Δ*t* = |*t* − *t*_0_| be the rescaled time. The “squares” and “circles” of Fig. 2(a) show the results for Δ*β* = 0.01 and 0.001, respectively, where *ρ*_*I*_ denotes the infected fraction. Two points can be noticed. The first one is that both of the two cases show the hysteresis loop in state space, denoted as *S*_Δ*t*_. The second one is that the value of *S*_Δ*t*_ for the case of Δ*β* = 0.01 is smaller than that of Δ*β* = 0.001, indicating that the decrease of Δ*β* will result in an increase of *S*_Δ*t*_. However, we find that there is a critical Δ*β*_*c*_ with *S*_Δ*t*_ = 0. After that, we always have *S*_Δ*t*_ = 0 for Δ*β* ≤ Δ*β*_*c*_. Thus, the system will have a hysteresis loop when Δ*β* Δ*β*_*c*_ but no hysteresis loop when Δ*β* ≤ Δ*β*_*c*_, indicating a phase transition between the states with and without hysteresis loop. This phenomenon can be explained as follows. For a fixed *T*, a smaller Δ*β* implies a smaller transient process for each updated *β*. When Δ*β* is small enough, the transient process will be less than *T*. In this situation, the time *T* will be long enough for the system of each updated *β* to reach its stationary state. Notice that the hysteresis loop comes from the adiabatical increase of *β* where *β* is updated before the system reaches its stationary state. Otherwise, there will be no hysteresis loop.

Secondly, we consider the case of fixing Δ*β* = 0.01. Figure 2(b) shows the results where the “squares” and “circles” represent the results of *T* = 1 and 5, respectively. Comparing them each other we see that the value of *S*_Δ*t*_ for the case of *T* = 1 is smaller than that of *T* = 5, indicating that *S*_Δ*t*_ increases with *T*. We also find that there is a critical *T*_*c*_ with *S*_Δ*t*_ = 0. The system will have a hysteresis loop when *T* < *T*_*c*_ but no hysteresis loop when *T* > *T*_*c*_, confirming again the phase transition between the states with and without hysteresis loop. The reason is that for a fixed Δ*β*, a larger *T* implies a smaller difference between the final state of each updated *β* and its stationary state. When *T* is large enough to make the difference disappear, we will have a zero *S*_Δ*t*_.

In sum, both the decrease of Δ*β* and the increase of *T* will make *S*_Δ*t*_ increase, indicating that they are the two key factors to influence the size of *S*_Δ*t*_. To see their relationship in details, Fig. 2(c) shows the dependence of *S*_Δ*t*_ on Δ*β* for fixed *T*, where the three curves with “squares”, “circles” and “triangles” represent the cases of *T* = 1, 2 and 3, respectively, and the inset shows the log-log plot. We see that *S*_Δ*t*_ decreases monotonously with the increase of Δ*β* and the three curves in log-log plot are approximately parallel each other, indicating that *S*_Δ*t*_ depends on Δ*β* by an approximate power law with a fixed scaling. Figure 2(d) shows the dependence of *S*_Δ*t*_ on *T* for fixed Δ*β*, where the three curves with “squares”, “circles” and “triangles” represent the cases of Δ*β* = 0.02, 0.01 and 0.005, respectively, and the inset shows the log-log plot. We see that *S*_Δ*t*_ increases monotonously with *T* and the three curves in log-log plot are also approximately parallel each other, indicating that *S*_Δ*t*_ depends on *T* also by an approximate power law with a fixed scaling.

**Figure 3:**
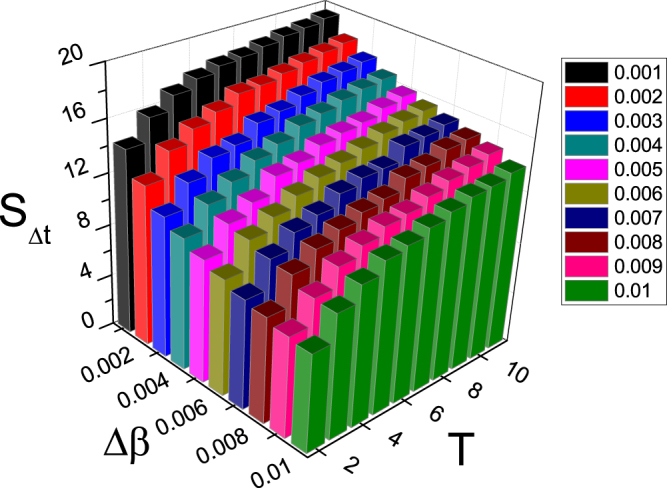
3D plot of the dependence of *S*_Δ*t*_ on the two parameters Δ*β* and *T* where the other parameters are the same as in Fig. 2(c) and (d).

To see the dependence of *S*_Δ*t*_ on Δ*β* and *T* more clear, Fig. 3 shows its 3D plot. We see that for each fixed *T*, the relationship between *S*_Δ*t*_ and Δ*β* is similar to the curves in Fig. 2(c). At the same time, for each fixed Δ*β*, the relationship between *S*_Δ*t*_ and *T* is similar to the curves in Fig. 2(d). Thus, *S*_Δ*t*_ is determined by the matching between Δ*β* and *T*.

**Figure 4:**
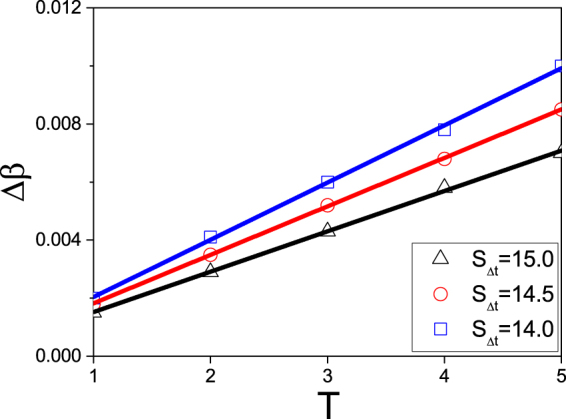
The relationship between Δ*β* and *T* for fixed *S*_Δ*t*_ where the curves with “squares”, “circles” and “triangles” represent the cases of *S*_Δ*t*_ = 14.0, 14.5 and 15.0, respectively.

It will be more interesting to study the relationship between Δ*β* and *T* when *S*_Δ*t*_ is fixed. Figure 4 shows the results where “squares”, “circles”, and “triangles” represent the cases of *S*_Δ*t*_ = 14.0, 14.5 and 15.0, respectively. We see that all the three cases are straight lines, indicating a linear relationship between Δ*β* and *T*. Thus, to keep *S*_Δ*t*_ unchanged, a larger Δ*β* needs a larger *T* to balance it, i.e. Δ*β* and *T* take the inverse role in sustaining the hysteresis loop.

### A paradox on the size of hysteresis loop between the state and parameter spaces

**Figure 5:**
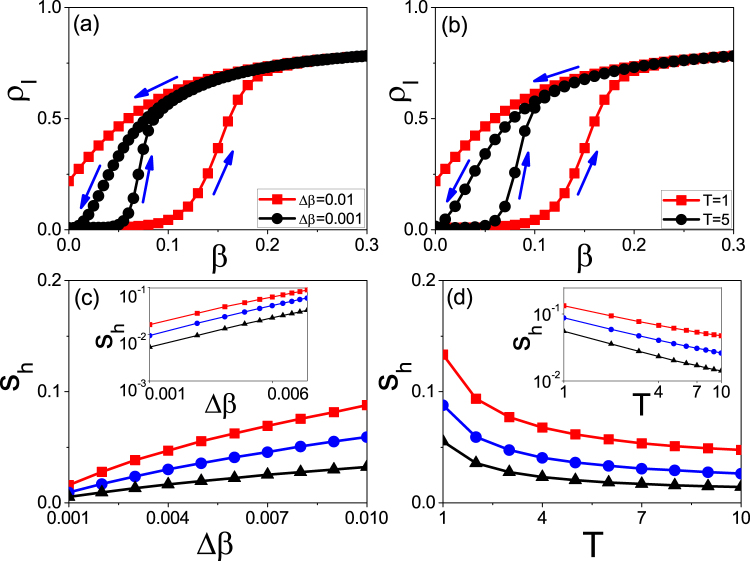
Hysteresis loops in parameter space where (**a–d**) corresponds to Fig. 2(a–d), respectively.

It is interesting to check the relationship between the state and parameter spaces. For this purpose, Fig. 5(a–d) show the results in parameter space corresponding to Fig. 2(a–d), respectively, where *S*_*h*_ represents the area of hysteresis loop in parameter space. From Fig. 5(a) we surprisingly find that the area *S*_*h*_ for the case of Δ*β* = 0.01 is larger than that of Δ*β* = 0.001, in contrast to the relationship of *S*_Δ*t*_ in Fig. 2(a). The similar situation has been observed in Fig. 5(b) where *S*_*h*_ for the case of *T* = 1 is larger than that of *T* = 5, which is also in contrast to the relationship of *S*_Δ*t*_ in Fig. 2(b). Therefore, we have observed two contradictory results of the same phenomenon that with the increase of Δ*β*, *S*_Δ*t*_ decreases in Fig. 2(c) but its corresponding *S*_*h*_ increases in Fig. 5(c), indicating a paradox that the size of hysteresis loop has an inverse dependence on the parameter Δ*β* between the state and parameter spaces. This paradox has been further confirmed by the case of increasing *T* where *S*_Δ*t*_ increases in Fig. 2(d) but its corresponding *S*_*h*_ decreases in Fig. 5(d).

To understand this paradox, we go back to the definitions of *S*_Δ*t*_ and *S*_*h*_. Let ${\rho }_{I}^{g}$ and ${\rho }_{I}^{c}$ be the infected fractions for the growing and recovering processes, respectively. Then, we have

2 $$\begin{array}{rcl}{S}_{{\rm{\Delta }}t} & = & \int ({\rho }_{I}^{c}(t)-{\rho }_{I}^{g}(t))dt\\  & = & \int ({\rho }_{I}^{c}(\frac{\beta }{{\rm{\Delta }}\beta }T+\delta t)-{\rho }_{I}^{g}(\frac{\beta }{{\rm{\Delta }}\beta }T+\delta t))dt\end{array}$$

where $\frac{\beta }{{\rm{\Delta }}\beta }$ denotes the integer of *t*/*T*, and *δt* is the fraction of *t*/*T* located in between 0 and *T*. Notice that *β* is a constant when *δt* changes from 0 to *T*. If we approximately use the average of ${\rho }_{I}^{g}$ and ${\rho }_{I}^{c}$ in the period from 0 to *T* to replace ${\rho }_{I}^{g}(t)$ and ${\rho }_{I}^{c}(t)$, Eq. (2) can be rewritten as

3 $${S}_{{\rm{\Delta }}t}\approx \int ({\rho }_{I}^{c}(\beta )-{\rho }_{I}^{g}(\beta ))\frac{T}{{\rm{\Delta }}\beta }d\beta =\frac{T}{{\rm{\Delta }}\beta }{S}_{h}$$

**Figure 6:**
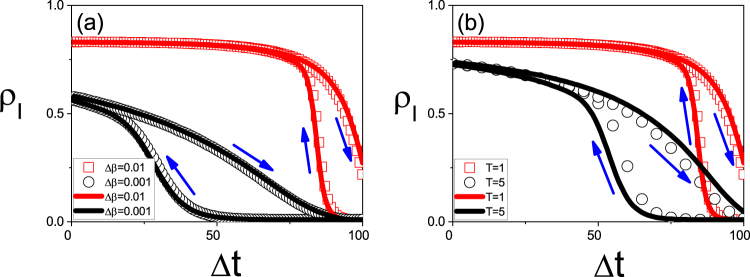
Consistence between the state and parameter spaces where the “squares” and “circles” in (**a**) and (**b**) come from Fig. 5(a) and (b) by Eq. (3), respectively, and the solid lines in (**a**) and (**b**) are the replotted Fig. 2(a) and (b), respectively.

Based on Eq. (3), we obtain a new quantity $t=\frac{T}{{\rm{\Delta }}\beta }\beta$ for each *β* in Fig. 5(a) and (b). Then, we transform all the *ρ*_*I*_(*β*) in Fig. 5(a) and (b) into their corresponding *ρ*_*I*_(*t*) and plot them in the new framework of *ρ*_*I*_(*t*) versus Δ*t*. The “squares” and “circles” in Fig. 6(a) and (b) show the results corresponding to that in Fig. 5(a) and (b) by Eq. (3), respectively. For comparison, we also replot the curves of Fig. 2(a) and (b) into Fig. 6(a) and (b) by the solid lines, respectively. Comparing the solid lines with their corresponding symbols in Fig. 6(a) and (b), respectively, we see that they are consistent each other, confirming the correctness of Eq. (3). Therefore, the paradox can be unified by Eq. (3).

**Figure 7:**
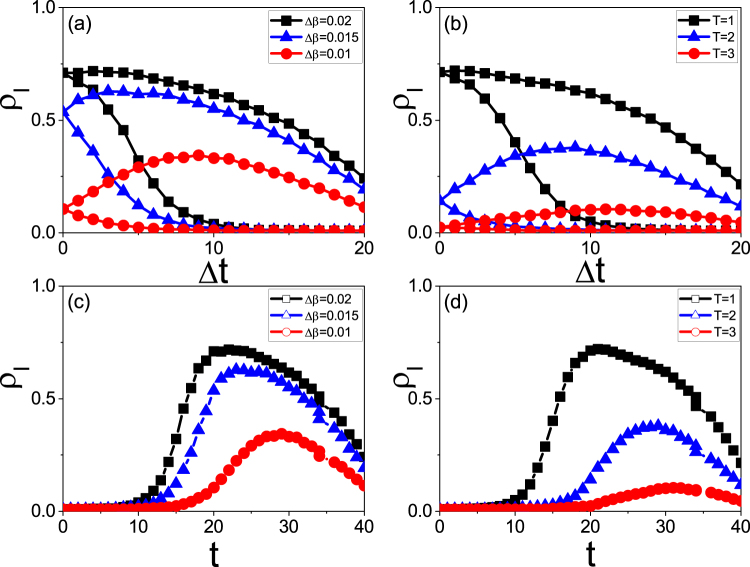
Case of fixed total evolutionary time as 2 × 20 (20 for the growing process and 20 for the recovering process) where all the parameters in (**a**) and (**c**) are the same as in Fig. 2(a) and that in (**b**) and (**d**) are the same as in Fig. 2(b). (**a**) and (**b**) represent the cases of *S*_Δ*t*_ for fixed Δ*β* and fixed *T*, respectively. (**c**) and (**d**) represent the cases of *S*_*t*_ corresponding to (**a**) and (**b**), respectively.

We have to point out that the evolutionary times of the two cases in Fig. 2(a) or (b) are different, implying that we have long enough time for the different cases to finish their hysteresis loop. However, this condition of long enough evolutionary time is not always guaranteed in realistic situations. For example, we notice from Fig. 1(a) that the time periods of different outbreaks are generally in the same level, i.e. a few months, although their *S*_*t*_ in Fig. 2(d) or *S*_Δ*t*_ in Fig. 2(e) may have significant difference. To incorporate this feature into simulations, we need to take the same evolutionary time for all the cases of Fig. 2(a) and (b). After considering this condition, Fig. 7(a) and (b) show the results corresponding to Fig. 2(a) and (b), respectively, where the evolutionary time for both the growing and recovering processes are fixed as *t*_*max*_ = 20. From Fig. 7(a) and (b) we see that their sizes of hysteresis loops can be large, small or even zero, depending on the values of Δ*β* and *T*. This result well explains the observation in Fig. 1(e), where the larger *S*_Δ*t*_ corresponds to a larger Δ*β* and a smaller *T* while the smaller *S*_Δ*t*_ corresponds to a smaller Δ*β* and a larger *T*. A zero *S*_Δ*t*_ can be expected when Δ*β* is smaller than Δ*β*_*c*_ or *T* is larger than *T*_*c*_, which corresponds to the background in Fig. 1(a). More important, we notice from Fig. 7(a) and (b) that *S*_Δ*t*_ has different relationship with Δ*β* and *T* from that in Fig. 2(a) and (b), indicating that the paradox disappear when the total evolutionary time is fixed.

Similarly, the fixed evolutionary time can be used to explain the different sizes *S*_*t*_ in Fig. 1(d). Figure 7(c) and (d) show the results for fixed evolutionary time *t*_*max*_ = 20, which correspond to Fig. 7(a) and (b), respectively. From Fig. 7(c) and (d) we see that *S*_*t*_ increases with Δ*β* and decreases with the increase of *T*, indicating that *S*_*t*_ has different behaviours with that of *S*_Δ*t*_ in Fig. 2(a) and (b). That is, the size of *S*_*t*_ is mainly determined by the outbreak of epidemic but not the asymmetry between the growing and recovering processes.

### A brief theoretical analysis

Based on the mean-field theory, we make a brief theoretical analysis for the above numerical results. For the SIS model, the evolution of *ρ*_*I*_ satisfies the following equation

4 $${\dot{\rho }}_{I}=\mathrm{(1}-{\mathrm{(1}-\beta (t))}^{{k}_{I}}){\rho }_{I}\mathrm{(1}-{\rho }_{I})-\mu {\rho }_{I},$$

where *k*_*I*_ represents the average number of infected neighbors of a node. During the evolutionary process, *k*_*I*_ will change with time. That is, *k*_*I*_ will gradually increase with *t* in the growing process but decrease in the recovering process. For convenience, we rewrite Eq. (4) as

5 $${\dot{\rho }}_{I}=\mathrm{(1}-{\mathrm{(1}-\beta (t))}^{{k}_{I}}-\mu ){\rho }_{I}-\mathrm{(1}-{\mathrm{(1}-\beta (t))}^{{k}_{I}}){\rho }_{I}^{2}$$

In the following, we will solve Eq. (5) for the growing and recovering processes, respectively.

#### The growing process

In this process, we have *β*(*t*) = *n*Δ*β* for *nT* < *t* < (*n* + 1)*T* and the initial condition *ρ*_*I*_(*t*) = *ρ*_*I*_(*nT*) for each updated *β*(*t*). Substituting them into Eq. (5) we have

6 $${\dot{\rho }}_{I}=\mathrm{(1}-{\mathrm{(1}-n{\rm{\Delta }}\beta )}^{{k}_{I}}-\mu ){\rho }_{I}-\mathrm{(1}-{\mathrm{(1}-n{\rm{\Delta }}\beta )}^{{k}_{I}}){\rho }_{I}^{2}$$

By dividing ${\rho }_{I}^{2}$ on both sides of Eq. (6), we have

7 $$\frac{d\frac{1}{{\rho }_{I}}}{dt}=\mathrm{(1}-{\mathrm{(1}-n{\rm{\Delta }}\beta )}^{{k}_{I}}-\mu )\frac{1}{{\rho }_{I}}-\mathrm{(1}-{\mathrm{(1}-n{\rm{\Delta }}\beta )}^{{k}_{I}})$$

Letting 1/*ρ*_*I*_ be a new variable, we can obtain the solution of Eq. (7) as

8 $${\rho }_{I}(t)=(1-\frac{\mu }{1-{\mathrm{(1}-n{\rm{\Delta }}\beta )}^{{k}_{I}}})\frac{1}{1+c{e}^{-\mathrm{(1}-{\mathrm{(1}-n{\rm{\Delta }}\beta )}^{{k}_{I}}-\mu )t}}$$

with *t* ∈ [*nT*, (*n* + 1)*T*]. Doing some simple operations, we have

9 $${\rho }_{I}(t)=(1-\frac{\mu }{1-{\mathrm{(1}-n{\rm{\Delta }}\beta )}^{{k}_{I}}})\frac{{c}_{1}{e}^{\mathrm{(1}-{\mathrm{(1}-n{\rm{\Delta }}\beta )}^{{k}_{I}}-\mu )t}}{{c}_{1}{e}^{\mathrm{(1}-{\mathrm{(1}-n{\rm{\Delta }}\beta )}^{{k}_{I}}-\mu )t}+1}$$

Letting *t* = *nT*, we obtain

10 $$\frac{1}{{c}_{1}}=(\frac{1-{\mathrm{(1}-n{\rm{\Delta }}\beta )}^{{k}_{I}}-\mu }{1-{\mathrm{(1}-n{\rm{\Delta }}\beta )}^{{k}_{I}}}\frac{1}{{\rho }_{I}(nT)}-1){e}^{\mathrm{(1}-{\mathrm{(1}-n{\rm{\Delta }}\beta )}^{{k}_{I}}-\mu )nT}$$

Substituting Eq. (10) into Eq. (9) and letting *t* = (*n* + 1)*T*, we obtain

11 $${\rho }_{I}((n+\mathrm{1)}T)=(1-\frac{\mu }{1-{\mathrm{(1}-n{\rm{\Delta }}\beta )}^{{k}_{I}}})\frac{{c}_{1}{e}^{\mathrm{(1}-{\mathrm{(1}-n{\rm{\Delta }}\beta )}^{{k}_{I}}-\mu )(n+\mathrm{1)}T}}{{c}_{1}{e}^{\mathrm{(1}-{\mathrm{(1}-n{\rm{\Delta }}\beta )}^{{k}_{I}}-\mu )(n+\mathrm{1)}T}+1}$$

#### The recovering process

In this process, we have *β*(*t*) = 1 − *n*Δ*β* for *nT* < *t* < (*n* + 1)*T*. Substituting it into Eq. (5) we have

12 $${\dot{\rho }}_{I}=\mathrm{(1}-{(n{\rm{\Delta }}\beta )}^{{k}_{I}}-\mu ){\rho }_{I}-\mathrm{(1}-{(n{\rm{\Delta }}\beta )}^{{k}_{I}}){\rho }_{I}^{2}$$

Similarly, we divide ${\rho }_{I}^{2}$ on both sides of Eq. (12) and obtain

13 $$\frac{d\frac{1}{{\rho }_{I}}}{dt}=-\,\mathrm{(1}-{(n{\rm{\Delta }}\beta )}^{{k}_{I}}-\mu )\frac{1}{{\rho }_{I}}+\mathrm{(1}-{(n{\rm{\Delta }}\beta )}^{{k}_{I}})$$

The solution of Eq. (13) can be obtained as

14 $${\rho }_{I}(t)=(1-\frac{\mu }{1-{(n{\rm{\Delta }}\beta )}^{{k}_{I}}})\frac{{c}_{1}{e}^{\mathrm{(1}-{(n{\rm{\Delta }}\beta )}^{{k}_{I}}-\mu )t}}{{c}_{1}{e}^{\mathrm{(1}-{(n{\rm{\Delta }}\beta )}^{{k}_{I}}-\mu )t}+1}$$

with *t* ∈ [*nT*, (*n* + 1)*T*]. Letting *t* = *nT*, we obtain

15 $$\frac{1}{{c}_{1}}=(\frac{1-{(n{\rm{\Delta }}\beta )}^{{k}_{I}}-\mu }{1-{(n{\rm{\Delta }}\beta )}^{{k}_{I}}}\frac{1}{{\rho }_{I}(nT)}-1){e}^{\mathrm{(1}-{(n{\rm{\Delta }}\beta )}^{{k}_{I}}-\mu )nT}$$

Substituting Eq. (15) into Eq. (14) and letting *t* = (*n* + 1)*T*, we obtain

16 $${\rho }_{I}((n+\mathrm{1)}T)=(1-\frac{\mu }{1-{(n{\rm{\Delta }}\beta )}^{{k}_{I}}})\frac{{c}_{1}{e}^{\mathrm{(1}-{(n{\rm{\Delta }}\beta )}^{{k}_{I}}-\mu )(n+\mathrm{1)}T}}{{c}_{1}{e}^{\mathrm{(1}-{(n{\rm{\Delta }}\beta )}^{{k}_{I}}-\mu )(n+\mathrm{1)}T}+1}$$

**Figure 8:**
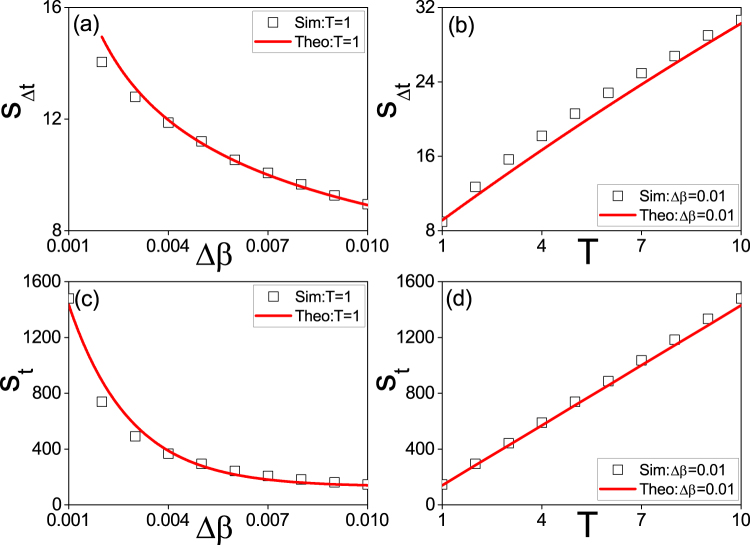
(**a**) and (**b**) represent the dependence of *S*_Δ*t*_ on the two parameters Δ*β* and *T* in state space, respectively, where the “squares” denote the numerical simulations and the solid lines are the theoretical results from Eqs (11) and (16). (**c**) and (**d**) represent the corresponding *S*_*t*_ of (**a**) and (**b**) in state space, respectively, where the “squares” denote the numerical simulations and the solid lines are the theoretical results from Eqs (11) and (16). The other parameters are the same as in Fig. 2(c) and (d).

So far, we have obtained the theoretical formulae Eqs (11) and (16) for the growing and recovering processes, respectively. Based on them, we can calculate ${S}_{{\rm{\Delta }}t}={\sum }_{t}({\rho }_{I}^{c}(t)-{\rho }_{I}^{g}(t))$, ${S}_{t}={\sum }_{t}({\rho }_{I}^{c}(t)+{\rho }_{I}^{g}(t))$, and their dependence on the two key parameters Δ*β* and *T*. The solid lines in Fig. 8(a–d) show the corresponding theoretical results, respectively. For comparison, we also put their corresponding numerical simulations, see the “squares”. We see that they are consistent with each other very well, indicating that the numerical results can be explained by the mean-field theory.

## Discussion

**Figure 9:**
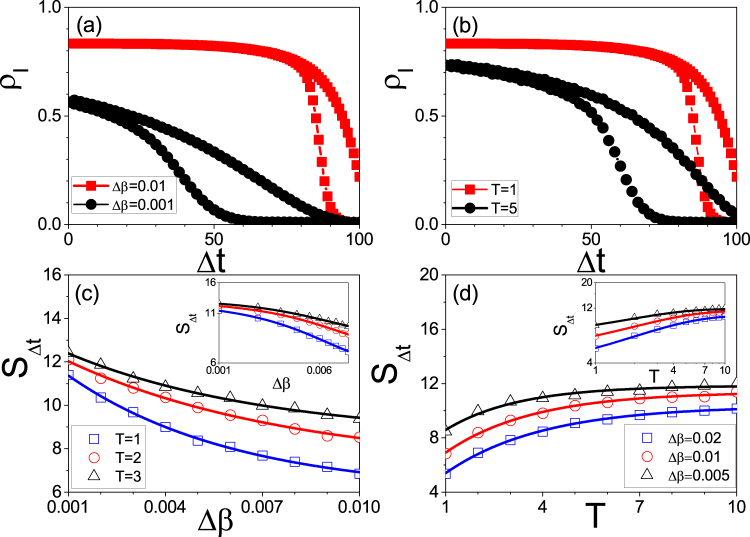
Hysteresis loops in state space for the case of SF network. (**a**) *ρ*_*I*_ versus Δ*t* for fixed *T* = 1 where the curves with “squares” and “circles” represent the cases of Δ*β* = 0.01 and 0.001, respectively. (**b**) *ρ*_*I*_ versus Δ*t* for fixed Δ*β* = 0.01 where the curves with “squares” and “circles” represent the cases of *T* = 1 and 5, respectively. (**c**) *S*_Δ*t*_ versus Δ*β* where the curves with “squares”, “circles” and “triangles” represent the cases of *T* = 1, 2 and 3, respectively. The inset shows the log-log plot. (**d**) *S*_Δ*t*_ versus *T* where the curves with “squares”, “circles” and “triangles” represent the cases of Δ*β* = 0.02, 0.01 and 0.005, respectively. The inset shows the log-log plot.

All the above results are based on the random ER network. Do they depend on the network topology? To check this robustness, we construct a scale-free (SF) network with the same size *N* = 10000 and the same average degree 〈*k*〉 = 6 as the ER network by the approach of ref.^52^. Based on this SF network, we have done the same process of numerical simulations as in the ER network and found the similar hysteresis loop and its dependence on the parameters Δ*β* and *T* with that of the ER network. Figure 9 shows the results, corresponding to Fig. 2. Comparing the corresponding panels between Figs 2 and 9, respectively, we see that they are all qualitatively similar to each other, confirming the robustness of the dependence of *S*_Δ*t*_ on Δ*β* and *T*.

**Figure 10:**
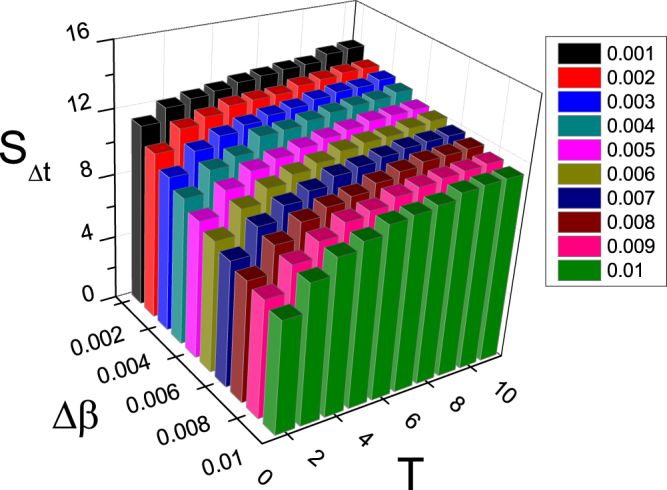
3D plot of the dependence of *S*_Δ*t*_ on the two parameters Δ*β* and *T* for the case of SF network where the other parameters are the same as in Fig. 9(c) and (d).

In detail, Fig. 10 shows the dependence of *S*_Δ*t*_ on both the parameters Δ*β* and *T* for the SF network. We see that for each fixed *T*, the relationship between *S*_Δ*t*_ and Δ*β* is similar to the curves in Fig. 9(c). At the same time, for each fixed Δ*β*, the relationship between *S*_Δ*t*_ and *T* is similar to the curves in Fig. 9(d). Thus, Fig. 10 confirms the result of Fig. 3 that *S*_Δ*t*_ is determined by the matching between Δ*β* and *T*.

We now understand that the outbreaks in recurrent epidemic data can be described by two ways. One is the size of outbreak, represented by *S*_*t*_. For this one, it is mainly determined by the values of *β* and *T*. The other is the size of hysteresis loop, represented by *S*_Δ*t*_, which is mainly determined by the matching of Δ*β* and *T*. Very interesting, we notice from Fig. 1(d) and (e) that there is a positive correlation between *S*_*t*_ and *S*_Δ*t*_, i.e. they show either larger or smaller values in most of the time. This correlation can be explained as follows. Because of the seasonal change of weather, the time period of epidemic outbreaks will be limited in a few months, see Fig. 1(a). Under this condition, a too small Δ*β* or too large *T* will not result in a larger accumulation of *β*, thus there will be no epidemic outbreak. With the increase of Δ*β* or decrease of *T*, the accumulation of *β* will increase to pass its critical value and thus result in an outbreak. At the same time, the increase of Δ*β* or decrease of *T* will make the system stay away from its steady state at each updated *β* and thus result in an increase of *S*_Δ*t*_. In this sense, *S*_*t*_ and *S*_Δ*t*_ can be unified in the same framework of Δ*β* and *T*, indicating that the hysteresis is a nature feature of epidemic outbreak in real case.

Basically, the existence of hysteresis loop is a memory effect from the adiabatical process. Without the adiabatical inheritance, for each concrete *β*, we will take random initial conditions for both the growing and recovering processes. In this way, the epidemic spreading will not have much difference between the growing and recovering processes for the case of either *T* > *T*_*c*_ or *T* < *T*_*c*_, indicating that the adiabatical process is one necessary condition for the hysteresis loop. Another necessary condition is that the parameter *β* has to be updated before the system reaches its steady state, i.e. *T* < *T*_*c*_; otherwise, there will no be difference between the growing and recovering processes when *T* > *T*_*c*_. Once these two conditions are satisfied, we will have the hysteresis loop. In details, for the case of adiabatical process with *T* < *T*_*c*_, its *ρ*_*I*_(*T*) will be less than the stabilized value of *ρ*_*I*_(*T*_*c*_) in the growing process as *β* is updated before *ρ*_*I*_(*T*) grows to its stabilization *ρ*_*I*_(*T*_*c*_), i.e. *ρ*_*I*_(*T*) < *ρ*_*I*_(*T*_*c*_). While in the recovering process, its *ρ*_*I*_(*T*) will be larger than *ρ*_*I*_(*T*_*c*_) as *β* is changed before *ρ*_*I*_(*T*) decreases to its stabilization, i.e. *ρ*_*I*_(*T*) *ρ*_*I*_(*T*_*c*_). This distinction causes the hysteresis loop for *ρ*_*I*_(*T*_*c*_). Thus, the area of hysteresis loop *S*_Δ*t*_ will be larger when the difference *T*_*c*_ − *T* is larger. On the other hand, the value of *T*_*c*_ depends on Δ*β*, i.e. a larger Δ*β* corresponds to a larger *T*_*c*_. Therefore, a larger *S*_Δ*t*_ will come from either a larger Δ*β* or a smaller *T*, which make a shorter transient process at each updated *β* and thus make the system stay away from its steady state. While a smaller *S*_Δ*t*_ will come from either a smaller Δ*β* or a larger *T*, which make the system close to its steady state at each updated *β*.

Moreover, it is necessary to say a few more words on the differences between this work and that of ref.^42^. The main contributions of ref.^42^ are two aspects: (1) the finding of asymmetry between the growing and recovering processes; and (2) the explanation form the angle of hysteresis loop. However, the former is from the state space while the later is from the parameter space, i.e. an indirect explanation. This work gives an explanation to the hysteresis loop directly from the state space and find a positive correlation between *S*_*t*_ and *S*_Δ*t*_. Moreover, we reveal a paradox between the state and parameter spaces and show a way to explain it. These findings imply that more attention should be paid to the features of epidemics in state space in the future, in contrast to the majority focus of epidemics from parameter space in previous studies.

In conclusion, we have studied the hysteresis loop mainly in the state space. Based on the SIS model, we find that there is a phase transition between the states with and without hysteresis loop in recurrent epidemics and the transition is controlled by two parameters, i.e. Δ*β* and *T*. The system will be in the state of no hysteresis loop when either Δ*β* is small enough or *T* is large enough, and in the state with hysteresis loop, otherwise. We also find a positive correlation between the size of outbreak and the size of hysteresis loop. It is shown that both *S*_*t*_ and *S*_Δ*t*_ depend on the parameters Δ*β* and *T* in power law, and Δ*β* and *T* take the inverse role for sustaining the hysteresis loop. Further, with the increase of *T* or the decrease of Δ*β*, a paradox of the area of hysteresis loop is observed, i.e. *S*_Δ*t*_ increases in the state space but *S*_*h*_ decreases in the parameter space. This paradox can be unified by Eq. (3). However, this paradox may not appear when the evolutionary time is fixed and not long enough. A theoretical analysis is given to explain the numerical simulations.
